# The Mechanical Treatment of Heart Disease

**Published:** 1896-06

**Authors:** 


### THE MECHANICAL TREATMENT OF HEART DISEASE.

The employment of systematic and carefully regulated muscular exercise in the treatment of cardiac disorders is of comparatively recent origin. While the method may never become universally applicable in the routine of general practice, there is, nevertheless, enough in it to merit serious attention.

Dr. Robert Saundby, of Birmingham, England, has published in the *British Medical Journal*, No. 1,818, 1895, some remarks on this system of treatment under the title of the "Schott [or Nauheim] Treatment of Heart Disease." Saundby's remarks bear impartial, testimony to the value of this method of practical therapy. The author tells us that the beneficial effects of Nauheim baths in the treatment of chronic cardiac disease were first proclaimed by the late Professor Benneke, about 1872, before which time there existed an unfouned dread of employing them in these cases. A few years afterward his teachings were reenforced by monograms from the pens of Drs. August and Theodor Schott, and the Nauheim bath treatment for heart disease gained considerable repute in Germany. The success obtained by the Zander movement cure induced Dr. A. Schott to devise a system of mild gymnastics or drill for the treatment of neurasthenic and hysterical patients, of whom a considerable number come annually to Nauheim.

Some of these patients had rapid weak hearts, and it was discovered with some surprise that the exercises, instead of quickening the heart's action, slowed and steadied it; further experience showed them that this result followed also in organic diseases of the heart, so that a method originally devised for steadying and strengthening the nervous system proved to be a valuable adjunct to the bath treatment of cardiac diseases.

As to the method of system followed at Nauheim, we are informed that it consists of medicinal baths and muscular exercises, supplemented if necessary by graduated mountain climbing.

Dr. Saundby is disposed to agree with Schott in the opinion that the system is of special value in all cases of chronic heart disease, except those in which there is advanced degeneration of the myocardium, or aneurism of the heart or great vessels, or advanced arterio-sclerosis. It has proved of great service in all forms of valvular disease, in congenital cardiac defects, in simple dilated hearts without valvular disease, in the functional cardiac debility of anæmia, however induced, in nervous irritable hearts, in simple tachycardia, and in the rapid heart of Graves' disease.

In regard to the exercises we learn that they are called "Widerstands-Gymnastik," or resistance gymnastics, and consist of slow movements executed by the patient and resisted by the physician or operator. A short interval is allowed after each movement, during which the patient sits down. The exertion employed must be very small, and should cause no increase in respiratory movements, flushing or pallor. The patient should be loosely and lightly clothed, and told to breathe quietly. The resistance made should be of such a kind that the patient may always feel himself easily the master. The operator must not grasp or in any way constrict the limb, but should oppose by the hand held flatly. The movements are nineteen in number.

1. Arms extended in front of body on a level with shoulder, hands meeting; arms carried out until in line, and brought back to original position.

2. Arms hanging at sides, palms forward; arm flexed at elbow until tips of fingers touch shoulder, back to original position; one arm only moved at a time.

3. Arms down, palms forward; arms carried outward and upward until thumbs meet over head; back to original position.

4. Hands in front of abdomen, fingers flexed so that second phalanges touch those of opposite hand; arms raised until hands rest on top of head; back to original position.

5. Arms down, palms against thighs; arms raised in parallel planes as high as possible; back to original position.

6. Trunk flexed on hips; return to original position.

7. Trunk rotated to left, right; return to original position.

8. Trunk flexed laterally.

9. As No. 1, but fists clenched.

10. As No. 2, but fists clenched.

11. Arms down, palms against thighs; each in turn raised forward and upward until arm is alongside of ear, then turned outward, and arm descends backward.

12. Arms down, palms to thighs; both together moved backward in parallel planes as far as possible without bending the trunk forward.

13. Thighs in turn flexed on trunk, opposite hand resting on chair.

14. Lower extremities in turn extended fully, and bent on trunk forward and backward to extreme limits of movement, opposite hand resting on chair.

15. Legs in turn flexed on thigh, both hands on chair.

16. Feet together; lower extremities in turn abducted as far as possible, and brought back to original position, opposite hand resting on chair.

17. The arms extended horizontally outward, are rotated from the shoulder-joint to the extreme limits forward and backward.

18. The hands in turn are extended and flexed on the forearm to extreme limits, and brought back in line with arm.

19. The feet in turn are flexed and extended to the extreme limits, and then brought back to their natural position.

Dr. Saundby says that these exercises are easily learned, and that nurses make very good operators, as they do not resist too much.

In conclusion the author holds that a system like Dr. Schott's, which has been slowly evolved and perfected by years of careful clinical work, comes to us claiming attention chiefly from its practical results, and is, in Saundby's opinion, in no way discredited by any differences or doubts as to the proper theory npon which it is based. The latter only becomes important when new developements of the system are started, based on doubtful theories, and lacking the support of his long experience.

And, finally, Dr. Saundby expresses his strong sense of the danger there is of checking the fair trial of this promising method by the hasty extension of it to cases forbidden by Dr. Schott, and against which he especially warns us. Worked carefully and conscientiously on the lines laid down by its originator, it seems capable of being usefully introduced into our hospitals, into private practice, and especially into the practice of physicians at watering-places, where many cardiac patients go annually for the treatment of their rheumatic and gouty ailments.—*N. Y. Medical Record.*

